# Chemotactic TEG3 Cells’ Guiding Platforms Based on PLA Fibers Functionalized With the SDF-1α/CXCL12 Chemokine for Neural Regeneration Therapy

**DOI:** 10.3389/fbioe.2021.627805

**Published:** 2021-03-22

**Authors:** Oscar Castaño, Ana López-Mengual, Diego Reginensi, Andreu Matamoros-Angles, Elisabeth Engel, José Antonio del Rio

**Affiliations:** ^1^Electronics and Biomedical Engineering, Universitat de Barcelona, Barcelona, Spain; ^2^Biomaterials for Regenerative Therapies, Institute of Bioengineering of Catalonia, Parc Cientific de Barcelona, Barcelona, Spain; ^3^CIBER en Bioingeniería, Biomateriales y Nanomedicina, CIBER-BBN, Madrid, Spain; ^4^Bioelectronics Unit and Nanobioeneering Laboratory, Institute for Nanoscience and Nanotechnology of the University of Barcelona, Barcelona, Spain; ^5^Molecular and Cellular Neurobiotechnology, Institute of Bioengineering of Catalonia, Parc Cientific de Barcelona, Barcelona, Spain; ^6^Department of Cell Biology, Physiology and Immunology, Faculty of Biology, Universitat de Barcelona, Barcelona, Spain; ^7^Centro de Investigación Biomédica en Red sobre Enfermedades Neurodegenerativas, Barcelona, Spain; ^8^Institute of Neurosciences, University of Barcelona, Barcelona, Spain; ^9^School of Medicine, Universidad de Panamá, Panama City, Panama; ^10^Biomedical Engineering Program, Universidad Latina de Panamá, Panama City, Panama; ^11^IMEM-BRT Group, Department of Materials Science, EEBE, Technical University of Catalonia (UPC), Barcelona, Spain

**Keywords:** olfactory ensheathing cells, electrospinning, PLA nanofibers, cell migration, gradients, SDF-1alpha, CXCL12

## Abstract

(Following spinal cord injury, olfactory ensheathing cell (OEC) transplantation is a promising therapeutic approach in promoting functional improvement. Some studies report that the migratory properties of OECs are compromised by inhibitory molecules and potentiated by chemical concentration differences. Here we compare the attachment, morphology, and directionality of an OEC-derived cell line, TEG3 cells, seeded on functionalized nanoscale meshes of Poly(l/dl-lactic acid; PLA) nanofibers. The size of the nanofibers has a strong effect on TEG3 cell adhesion and migration, with the PLA nanofibers having a 950 nm diameter being the ones that show the best results. TEG3 cells are capable of adopting a bipolar morphology on 950 nm fiber surfaces, as well as a highly dynamic behavior in migratory terms. Finally, we observe that functionalized nanofibers, with a chemical concentration increment of SDF-1α/CXCL12, strongly enhance the migratory characteristics of TEG3 cells over inhibitory substrates.

## Introduction

Traumatic injuries to the central nervous system (CNS) are associated with the loss, in most cases, of crucial physiological functions. Spinal cord injury (SCI) is, unfortunately, a prime example, since it substantially affects the quality of life through loss of function (paralysis and anesthesia) and development of pain and secondary disabilities. Many biological limitations restrict the efficacy of current SCI repair strategies, and many of the regenerative mechanisms are impaired in patients with neuropathies, further limiting their recovery (Silver et al., 2014; Pounders et al., 2019). As a consequence, although there have been advances [i.e., (Reichert, 2007)], repair of the damaged spinal cord continues to be a major paradigmatical challenge in regenerative medicine. Neural tissue engineering offers hope to patients and is a rapidly growing field aimed at creating engineered tissue able to replace dead tissue in affected regions and recover lost function (Akter, 2016).

Olfactory ensheathing cells (OECs) play a key role in the guidance of olfactory axons by trophic and physical support (Kafitz and Greer, 1999; Sonigra et al., 1999). These properties have led to their implementation in several cell therapeutical approaches in the damaged CNS. Several studies have demonstrated beneficial effects following OEC transplantation in animals after SCI. Evidence of anatomical regeneration (Ramón-Cueto and Nieto-Sampedro, 1994; Li et al., 1997; Imaizumi et al., 2000; Nash et al., 2002) and functional improvement (Li et al., 1997; Ramón-Cueto et al., 1998, 2000; Lu et al., 2001, 2002; López-Vales et al., 2007) has been observed in a variety of SCI models, including in hemisection and in complete transection of the spinal cord.

Olfactory ensheathing cell transplantation is a cellular alternative therapeutic approach for SCI. In general, five beneficial effects of OECs have been reported that can promote functional recovery: (i) stimulation of axonal growth; (ii) tissue and axon preservation; (iii) capability to mix intimately with healing glia; (iv) promotion of angiogenesis; and (v) capability to ensheath growing axons [i.e, see Roet and Verhaagen (2014) for review]. However, therapy based on the use of OECs has a series of problems that leads to variable and contradictory results, mostly caused by the inherent complexity of the regenerative process and the myriad variables involved [e.g., receptors, ligands (inhibitory substrates)] (Field et al., 2003; Thuret et al., 2006; Gómez et al., 2018). In addition, unfortunately, transplanted OECs show a high rate of cell death in the host as a result of the excitotoxicity associated with the lesion, in the primary as well as the penumbra regions (Torres-Espín et al., 2014), almost disappearing 2–3 weeks after transplantation. Therefore, the number of cells per transplant must be high (≈ 200,000 cells; Guntinas-Lichius et al., 2001; Tabakow et al., 2013). Furthermore, transplanted OECs showed a low degree of migration in the lesioned spinal cord (Reginensi et al., 2015).

In the primary olfactory system, OECs migrate from the periphery (olfactory mucosa) into the CNS (olfactory bulb), and this organized migration is also able to enhance axonal extension after injury of the olfactory tract (Ekberg et al., 2012). However, as noted above, the migratory properties of OECs are extremely limited in the area of injury in the spinal cord due to the presence of inhibitory molecules in the extracellular environment (Gudiño-Cabrera and Nieto-Sampedro, 2000; Deng et al., 2006). Several factors have been described as modulators of the migration of OECs, including glial-derived neurotrophic factor (GDNF), fibulin-3, slit-2, myelin-associated inhibitors (MAIs; e.g., Nogo-A, MAG, or OMgp), and chondroitin sulfate proteoglycans (CSPG; Cao et al., 2006; Su et al., 2007; Vukovic et al., 2009; Huang et al., 2011; Nocentini et al., 2012; Reginensi et al., 2015). Most of these molecules inhibit the migration of OECs, and they are overexpressed in the meningo-glial scar after injury, acting as negative players in the migration of transplanted OECs.

For this reason, researchers seek to enhance the regenerative potential of OECs in combination with other treatments, for example, gene therapy, in order to obtain a local, continuous release of factors that improve axonal growth, neutralizing factors of the inhibitory signal present in the injured environment, and biomaterials to allow guided targeting and better structural support in the area of injury. In previous studies, we analyzed the migratory properties and biomechanical aspects of OECs growing on inhibitory substrates, such as MAIs (Nocentini et al., 2012; Reginensi et al., 2015), or CSPGs (Reginensi et al., 2015). We evidenced that in the presence of inhibitory molecules, the migratory capacity and traction force of OECs are drastically reduced, which correlates with profound changes in the F-actin cytoskeleton and the distribution of focal adhesion complexes (Nocentini et al., 2012; Reginensi et al., 2015). As noted above, several studies using ducts made with biomaterials have been reported with differing levels of success in regenerative strategies. These biomaterials, both natural and artificial, must meet a series of specific requirements. Natural biomaterials, in general, are difficult to handle due to their low mechanical resistance, while artificial materials stand out for their low bioactivity and signaling control to properly guide cells or lesioned axons in the repair process (Chen et al., 2018). Nanobiotechnology has been able to manufacture biodegradable materials at a controlled submicron scale using the technique of electrospinning to create nanofibers mimicking the extracellular matrix (ECM). The method is very versatile and allows for positioning of the fibers with relative precision. Electrospinning offers a topography that cells can easily recognize (Álvarez et al., 2013, 2014; Oliveira et al., 2016) and also provides a high-surface area-to-volume ratio that can be modified and functionalized with a bioactive group or signals to enhance cell adhesion, morphology, and fate control (Ghasemi-Mobarakeh et al., 2008; Beachley and Wen, 2009; Hackett et al., 2010). However, there have been few successful studies of the production of biocompatible matrices for the promotion of cell migration and axonal regrowth in models of CNS injury (Straley et al., 2010).

A significant field of investigation in SCI is the application of these biomaterials to perform as guiding supports at the place of damage and/or the application as a matrix for the release of signaling factors to encourage nerve regeneration (King et al., 2010). For example, fibers and scaffolds based on materials such as poly (lactic-co-glycolic acid; PLGA), Poly(l/dl-lactic acid; PLA), and NeuroGel (methacrylamide-based hydrogel) have shown an encouraing positive result in terms of functionality when used as support for axon growth. Additionally, gradients of biomolecules on synthetic biomaterials can efficiently mimic the natural, graded variation of properties with the ECM. Such gradients represent accessible study boards for improving understanding of cellular activities, and they also provide functional support for tissue engineering. The created morphological gradients regulate the settlement of cells, diffusion of nutrients, and removal of cellular waste products during cell proliferation and differentiation, while also providing a progressive variation of mechanical properties within the overall support mechanism.

The combination of electrospinning techniques in the generation of nanofibers based on synthetic materials (e.g., PLA, PLGA), functionalized with bioactive molecules that act with a chemical gradient (e.g., SDF-1α, TNF-α) for certain biological systems, may be relevant for the development of new approaches to tissue engineering. TEG3 cells are an immortalized cell line of OECs that preserve the pro-regenerative features of primary cells (Moreno-Flores et al., 2003), and they also express the C-X-C chemokine type 4 receptor (CXCR-4) with a high affinity to the chemokine stromal cell-derived factor 1 (SDF1alpha also known C-X-C motif chemokine 12, SDF-1α/CXCL12) triggering intracellular signaling processes mediated by several kinases [i.e., Akt and extracellular signal-regulated kinase (pERK)1/2] (Levoye et al., 2009).

We hypothesize that less rigid and more amorphous topographies, such as PLA (PLDLA 80/20) nanostructured fibers, functionalized on their surface with different concentrations of a chemotactic agent, would guide the migration of TEG3 cells toward more chemokine-concentrated surfaces. We then modeled a damaged gap by creating an inhibiting microenvironment using a growth inhibitory molecule as the CSPG, in contrast to the adhesive permissive laminin coatings. The bridging system was designed and optimized using PLA electrospun fibers, which were functionalized with a chemotactic agent such as the SDF-1α/CXCL12 chemokine to get an *in situ* increment of migration signaling on the surface to drive cells through the fibers.

## Materials and Methods

### Antibodies and Biochemicals Reagents

The reagents used for coating treatments were Poly-*L*-Lysine (P4707; Sigma-Aldrich, Merck Life Science), Laminin (L2020; Sigma-Aldrich, Merck Life Science), CSPG (CC117; Millipore, Merck Life Science), Phalloidin-TRITC (P5282; Sigma-Aldrich, Merck Life Science), diamidino-2-phenylindole (DAPI; B2261; Sigma-Aldrich, Merck Life Science), Rhodamine B (R6626, Sigma-Aldrich, Merck Life Science), SYLGARD^TM^ 184 Silicone Elastomer Kit (1673921; Dow Corning, Belgium), and recombinant murine SDF-1α (CXCL12; 250–20A; Peprotech).

The primary antibodies used in immunocytochemistry were SDF-1α antibody (Abcam; AB25117), b3 integrin (MAB2514; Millipore, Merck Life Science), Vinculin (Abcam, ab155120), and green fluorescence protein (GFP; A11122; Invitrogen^TM^, Thermo Fisher Scientific, Waltham, MA, United States). The secondary antibodies used were Alexa Fluor 488 donkey anti-rabbit (A21206; Invitrogen^TM^, Thermo Fisher Scientific, Waltham, MA, United States) and Alexa Fluor 488 goat anti-rat (A11006; Invitrogen^TM^, Thermo Fisher Scientific, Waltham, MA, United States).

### TEG3 Cultures

The immortalized clonal cell line TEG3 was used in this study. These are rat OEC primary cultures transfected with the simian vacuolating virus 40 (SV40) large *T* antigen (Moreno-Flores et al., 2003). In the study we used the original TEG3 cell line and a modified TEG3 cell line that expressed the enhanced green fluorescent protein (eGFP; Reginensi et al., 2015). Cells were maintained in Dulbecco’s Modified Eagle Medium/Nutrient Mixture F-12 (DMEM–F12, 11320033; Invitrogen^TM^, Thermo Fisher Scientific, Waltham, MA, United States) supplemented with 10% bovine calf serum (12133C; Sigma-Aldrich, Merck Life Science), 20 μg/ml pituitary extract (13028014; Invitrogen^TM^, Thermo Fisher Scientific, Waltham, MA, United States), 2 μM forskolin (F6886; Sigma-Aldrich, Merck Life Science), 1% penicillin-streptomycin (15140122; Invitrogen^TM^, Thermo Fisher Scientific, Waltham, MA, United States), and 1% fungizone (15290026; Invitrogen^TM^, Thermo Fisher Scientific, Waltham, MA, United States). TEG3 cells between passages 4–8 were used for the experiments.

### Culture Surface Coating and Immunocytochemical Methods

Glass coverslips (12 mm Ø) were coated essentially as described (Nocentini et al., 2012; Reginensi et al., 2015). Briefly, coverslips were pre-coated with Poly-*L*-Lysine 10 μg/ml dissolved in 0.1 M phosphate-buffered saline solution (PBS, pH 7.3) and then washed. After rinsing, they were coated with laminin (2 mg/ml, dissolved in 0.1 M PBS) and washed twice with 0.1 M PBS. In inhibition experiments, CSPG (20 μg/ml) was added instead of Laminin. TEG3 cells were cultured for 20 h and then the coverslips were fixed in 4% buffered paraformaldehyde for 30 min, permeabilized with 0.1% Triton X-100, and blocked with 10% fetal bovine serum (FBS), both diluted in 0.1 M PBS. Cells were sequentially incubated overnight with primary antibodies at 4°C and with the corresponding Alexa Fluor-tagged secondary antibodies for 1 h at room temperature. After rinsing in 0.1 M PBS, cells were stained with 0.1 M 4′,6- DAPI diluted in 0.1 M PBS for 10 min, rinsed, and mounted on Fluoromount^TM^ (Vector Labs, Burlingame, CA, United States); they were then analyzed using an Olympus BX61 fluorescence microscope equipped with a DP12L cooled camera or a Zeiss LSCM 500 confocal microscopy. Digital image processing was performed with ImageJ^TM^ software.

### Fabrication of PLA Nanofibers Using Electrospinning

Poly(l/dl-lactic acid; PLA, PURASORB^®^ PLDL 8038 Corbion, Amsterdam, Netherlands, inherent viscosity midpoint 3.8 dl/g) fibers were produced by electrospinning onto square windows of Parafilm^TM^ (Bemis Company, Inc, Neenah, WI, United States) hollow frames (12 mm × 12 mm inner window; 15 mm × 15 mm outer frame) and cover slides (15 mm diameter × 0.1 mm thick) attached to a rotary collector using adhesive tape. Cover slides were previously washed with soap and water and then cleaned in water, acetone, and methanol for 10 min in an ultrasound bath. Several concentrations (3, 4, 6, 8, and 9% w/w in 2,2,2-trifluoroethanol; 99.8%; Panreac, Barcelona, Spain) were tested to optimize the fiber thickness. Rhodamine B 0.01% related to PLA was added to the solutions to stain the fibers. The electrospinning process took just 30 s at 14 kV, 20 cm tip-collector distance, 1,200 rpm rotary speed, and the ambient humidity was kept at RH = 30% at 16°C. In the spinning process of the PLA-aligned nanofibers (Álvarez et al., 2014), the fibers were oriented perpendicular to the permissive (laminin) and inhibitory (CSPG) coatings and attached to coverslips in sterile conditions.

### Manufacture of PLA Biofunctionalized SDF-1α/CXCL12 Nanofibers for *in situ* Surface Concentration Increments

Both fibrous frames and fibrous coated cover slides with PLA nanofibers (diameter, 950 nm) were functionalized with SDF-1α/CXCL12 (Peprotech) chemokine using a dip-coating method to obtain a surface concentration difference. Briefly, fiber surfaces were first hydrolyzed for 10 min with a 0.01 M sodium hydroxide (NaOH) solution. After rinsing in pure water, they were immersed in an MES pH = 5.5 buffered solution of 1-ethyl-3-(3-dimethylaminopropyl)carbodiimide/N-hydroxysuccinimide (EDC/NHS) 1/1.2 for 10 min. Afterward, fibers were again rinsed and dip-coated in a solution of SDF-1α/CXCL12 of 50 ng/ml at a speed of 10 mm/min. Fibers were then rinsed again and store for further assays.

### Mechanical Characterization of PLA Fibers

The mechanical assessment was performed by uniaxial tensile-strain Zwiki Z0.5TN (Zwick-Roell, Ulm, Germany) analysis parallel with the direction of the fibers. Fibers were electrospun following the same conditions as section “Fabrication of PLA nanofibers using electrospinning” but for 3 h, yielding a mat of about 20–30 μm thickness in the center of the aluminum foil used to collect fibers. Then samples were cut following an ISO 527-1 standard with a bone shape. Then the bone-shaped mat was wrapped to form a cylinder that was coupled to the tensile-strain grips. The cell-load used had a maximum of 5N. The section was assessed by measuring the half-width of the cylinders using a high precision digital Mitutoyo micrometer 293–344 (Mitutoyo, Kanagawa, Japan). Measurement was performed at a speed of 10 mm/min until rupture. Elastic or Young’s modulus was approached by linear regression of the linear area of the elastic area.

### Crystallinity Content (χc) and Glass Transition Temperature (T_g_) of the PLA Fibers

Thermal features were assessed using differential calorimetric analysis (DSC, Q20, TA Instruments, Waters, DE, United States). 5 mg of fibers were encapsulated in aluminum pans and held to a thermal treatment between room temperature and 200°C at a 10°C/min rate for 2 cycles under N_2_ atmosphere. Degree of crystallinity was obtained following the relation%χc = (ΔH_m_–ΔH_c_)/ΔH^0^_m_, where**%**χc is crystallinity content expressed as a percentage, ΔHm is the latent melting point, and ΔHc is the heat of the crystallization, both obtained integrating the corresponding DSC peaks, and ΔH^0^_m_ is the melting point of PLA with an assumed degree of crystallinity of 100%. This has a value of 93.1 J/g (Álvarez et al., 2013).

### Morphological Characterization of PLA Fibers and Fixed Cells

Micro-and nano-morphology of PLA was assessed using field emission scanning electron microscope (FESEM, Nova^TM^-Nano SEM-230; FEI Co., Hillsboro, OR, United States) operating at 5.00 kV. Before imaging, samples were coated with an ultra-thin carbon layer to improve conductivity. Mean fiber diameter was measured considering at least 25 randomly selected fibers and using the ImageJ^TM^ analysis software (Schneider et al., 2012), and quantification of the fibers directionality was assessed using Fiji open-source platform (Schindelin et al., 2012) and its directionality plugin. Images of fibers and cells were produced by fixing cells with a solution in paraformaldehyde at 4% (PFA, Electron Microscopy Sciences, United States) for 15 min after rinsing cells twice with sterile 1 × PBS. Then they were washed again with 1 × phosphate-buffered saline (PBS).

As PLA fibers cannot be immersed in ethanol solutions due to the damage caused by alkoholisis, we decided to freeze them gradually, first at 4°C, then a −20°C, and finally at −80°C, followed by a process of lyophilization for 12 h. Afterward, they were coated with an ultra-thin layer of gold.

### Circularity Index and Morphological Cell Analysis

Coverslips were coated with Laminin (control) or CSPG (inhibitory substrate). 3 × 10^4^ TEG cells were seeded in coverslips with nanofibers for *in vitro* experiments. For cell imaging, thresholded images are previously pretreated with a bandpass filter and are subjected to circularity analysis. The index circularity with a value of 1.0 indicates a cell with rounded morphology, while as it approaches 0 it indicates that the cell has a bipolar structure based on the formula: 4π × (Area/Perimeter^2^). The digital analysis process was performed using ImageJ^TM^ software.

### Time-Lapse Analysis of TEG3 Migration Over PLA Nanofibers

For time-lapse analysis, Fluorodish cell culture dishes (World Precision Instruments, Sarasota, FL, United States) were coated with Poly-L-lysine and then with Laminin or CSPG as indicated above. Next, PLA nanofibers (functionalized or not) were placed over the coverslip in a parallel orientation. In order to analyze cell/fiber interaction and migration, we placed a polydimethylsiloxane (PDMS) membrane with a 500-μm-wide rectangular opening on top of the coated plate and PLA fibers. Next, 2 × 10^4^ TEG3 cells were seeded in the PDMS hole for 12 h to improve cell attachment, and the PDMS mask was gently removed, preserving the PLA fibers and allowing TEG cells to interact with fibers or the substrate while avoiding detached cells. TEG cells were cultured, and the time-lapse analysis was performed. In some cases, cultures were fixed after different time points (4 and 6 days) and immunochemically processed as above. For live imaging, culture dishes were transferred to a Live Cell Instruments system (LCI Instruments, Seoul, South Korea) for 25 h. Tracking was performed with an inverted Olympus microscope IX71 (20X objective), and images (5 megapixels each) were captured with either an ORCA Flash 4 or CX50 Olympus camera (17 fields, 190 frames each field, one frame every 8 min. 25 h in total; Nocentini et al., 2012; Reginensi et al., 2015). Images were then compiled as single image stacks and exported as uncompressed Audio Video Interleave (AVI) video files. The migration of TEG3 cells was analyzed using the MTrack plug-in from the ImageJ^TM^ software.

### Statistical Analysis

Quantitative data are expressed as mean ± S.E.M. (standard error of the mean) of at least three independent experiments. Means were compared with a one-way ANOVA test. *A* value of *p* < 0.05 was considered statistically significant.

## Results

### TEG3 Cytoskeletal Dynamics and Cell Morphology

**FIGURE 1:**
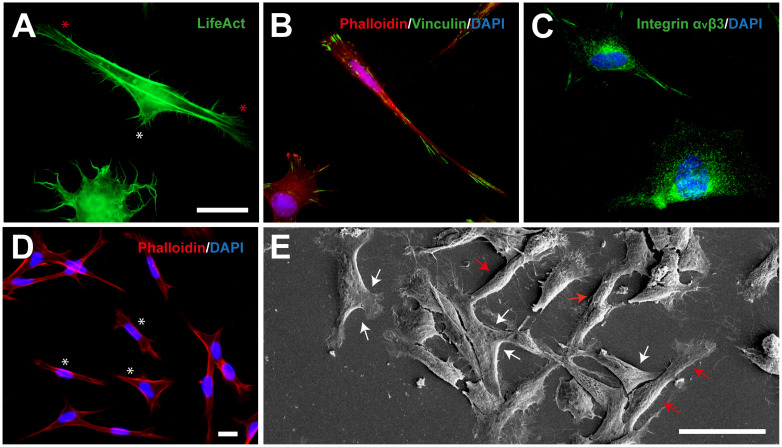
TEG3 cytoskeletal dynamics and phenotype morphology. **(A)** Lentiviral transfected LifeAct-eGFP TEG3 showing a high expression of membrane protrusions through the presence of motile lamellipodia; trailing process (white asterisk) and in the cell body (red asterisk); **(B)** intracellular distribution of *F*-actin and focal adhesions (FAs) on laminin substrate; **(C)** stain of integrin αvβ3; **(D)** flattened TEG3 cells showing membrane protrusion on one or both sides; and **(E)** FESEM image of a mix of bipolar (Schwann-type OECs, red arrows) and flattened (astrocyte-type OECs, white arrows) cells under *in vitro* conditions. Scale bars **(A–C)** – 10 μm; **(D)** – 10 μm; and **(E)** – 50 μm.

TEG3 is a clonal OEC line that shows similar growth-promoting potential to non-modified OECs (Moreno-Flores et al., 2003). In the first experiments, we seeded TEG3 cells in a permissive substrate (Laminin) to study their cytoskeletal characteristics and phenotype morphology. Lifeact, a small peptide with an affinity for actin microfilaments, has become one of the gold standards in cell imaging of actin, especially in cell morphology (Flores et al., 2019). Lentiviral transfected LifeAct-eGFP TEG3 cells showed high expression of membrane protrusions through the presence of motile lamellipodia, both in their trailing process and in the cell body (white and red asterisk, respectively, in Figure 1A). Also, we determined the presence of focal adhesions (FAs; vinculin-positive) on the laminin-coated substrate. TEG3 cells adopted two different morphologies as represented by the F-actin (Phalloidin-TRITC) distribution (Figure 1B) as well as the presence of integrin αvβ3 (Figure 1C). This indicates that TEG3 presents an active and dynamic mechanotransduction system able to sense physical properties (i.e., substrate stiffness) of the surrounding environment and thus modulate adhesion and traction forces with the ECM. Also, as described in other studies (Aguzzi et al., 2013; Reginensi et al., 2015), we observed the presence of two phenotype subpopulations of cultured OEC-TEG3 cells: bipolar morphology [Schwann cell-like OEC (sOEC-TEG3)] (red arrows), and flattened morphology [astrocyte-like OEC (aOEC-TEG3)] with planar stellate morphology (white arrows; Figures 1D,E). F-actin distribution showed the morphological plasticity of TEG3 cells. The bipolar TEG3 cells tend to have a single leading process and extensive trailing process (red arrows), while the flattened TEG3 cells tend to have a large membrane protrusion from one or both sides (Figure 1D). Also, using high-resolution microscopy (FESEM) we observed what it seems a mix of bipolar (Schwann-like OEC-TEG3 cells) and flattened (astrocyte-like OEC-TEG3 cells) under *in vitro* conditions (Figure 1E).

### Cytomorphometric Analysis and Cell Tracking of TEG3 Over PLA Nanofibers

**FIGURE 2:**
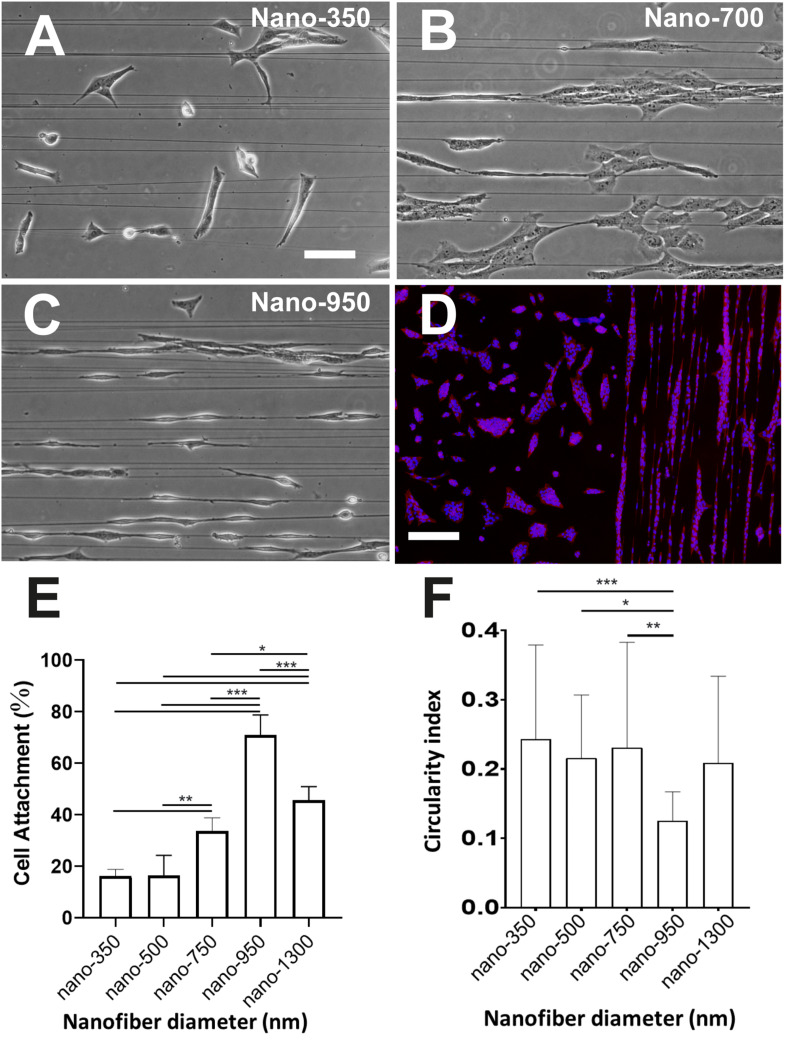
Cytomorphometric analysis of TEG3 over different diameter PLA nanofibers. Cells adhered onto fibers on coverslips with associated laminin of an averaged fiber diameter of **(A)** 350 nm, **(B)** 700 nm, and **(C)** 950 nm; **(D)** DAPI/phalloidin fluorescent image of cells adhered to a surface with (right) and without (left) 950 nm-fibers over an inhibiting coating of CSPG; **(E)** histogram showing the percentage of attachment of the cells with the different diameters of PLA nanofibers (biomaterial; ***p-value < 0.0001; **p-values 0,0017 and 0.0020; *p-value 0.0411; and *N* = 5); and **(F)** their shape circularity index of cells attached to the different fibers assessed in this work (***p-value < 0.0002; **p-value 0.0019; *p-value 0.0170; and *N* > 25). Scale bars **(A)** (same for **B,C**) – 50 μm; and **(D)** – 300 μm.

Electrospinning was applied to create aligned fiber meshes with differing average fiber diameters. Fibers were satisfactorily produced using PLA 80LL/20DL isomeric copolymer ratios, and the control of the thickness was reproducible by modifying the electrospinning solution concentration and viscosity. Fibers were slightly crystalline according to DSC measurements (%χ_c_ = 10.2%) with a Tg of 59.1°C, and mechanical tensile-strain assessment showed maximum stress of 97.4 ± 34.3 MPa and an approached elastic modulus of 2.2 ± 1.0 GPa. Fiber diameters were measured from FESEM micrographs using ImageJ^TM^ software. A minimum of 30 fibers was measured to obtain the average fiber diameter of each sample. TEG3 cells were seeded at low-density ratio on coverslips coated with laminin together with PLA nanofibers of various diameters. Several thicknesses were prepared in the range between 300 and 1,400 nm (Figures 2A–C). A higher cell attachment was found for 950 nm fibers (Figure 2E). In cell imaging, different shape descriptors can measure the perimeter, area, length, and roundness of individual cells, once an ellipse is fitted over a given cell. Circularity index is a normalized ratio of area to the perimeter (1 for a circular shape and 0 for a linear shape) and, biologically, lower circularity values describe elongated cell over the fibers. According to our cell morphology analysis, TEG3 cells have the lowest circularity index of 0.13 ± 0.04 when cultured on 950 nm fibers (Figure 2F). In PLA nanofibers, with a diameter of 950 nm., the cells have an elongated, bipolar-type structure associated with deformation of the cytoskeleton, in alignment with the direction of nanofibers. Furthermore, the rest of the PLA fiber diameters (350, 500, 750, and 1,300 nm) present a higher index of circularity over 0.2 (350 nm = 0.24 ± 0,14; 500 nm = 0.22 ± 0.09; 700 nm = 0.23 ± 0.15; and 1,300 nm = 0.21 ± 0,13) which indicates that the TEG3 cells are more irregular and anysotropic, and they have less presence of membrane protrusions, as well as a trailing process of cell-biomaterial interaction (Figure 2D).

**FIGURE 3:**
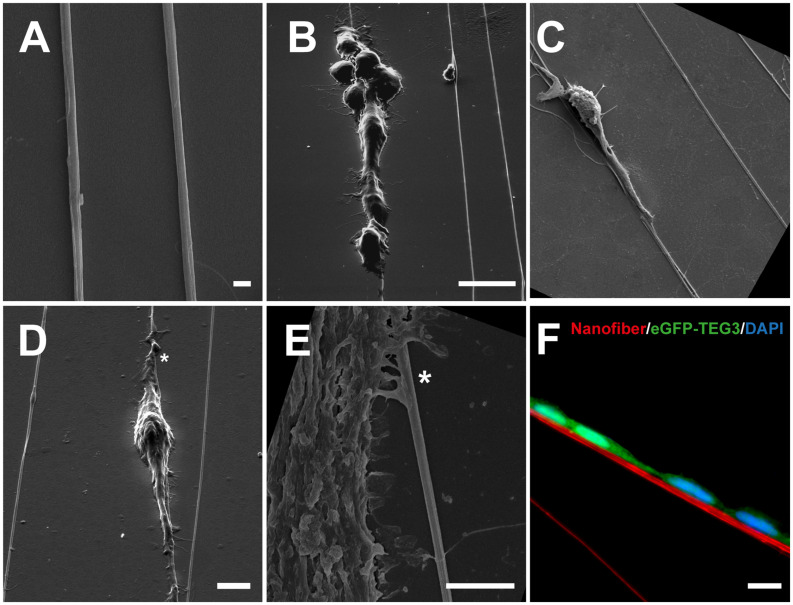
FESEM analysis of TEG3 over PLA nanofibers of differing diameters. FESEM images of **(A)** pure nanofibers of an average size of 950 nm aligned on coverslips with a laminin coating; **(B, C)** analysis of the cell morphology on the fibrous substrates showing that cell bodies are elongated along the axis of the fiber and extended trailing processes that are guided by fiber directionality; **(D)** peripheral lamellipodial waves bearing fine filopodia showing cell-cell contact and cell group migration over the nanofibers; **(E)** cell membrane on the migratory front interacting with the nanotopography of PLA nanofiber (asterisk); **(F)** detail of the elongation of eGFP-TEG3 over a stained fiber (red) with immunofluorescence techniques. Scale bars: **(A)** – 1 μm., **(B)** (same for **(C)**) – 50 μm; **(D)** – 5 μm; **(E)** – 7.5 μm; and **(F)** – 10 μm.

Aligned fibers were achieved by targeting the charged polymer jet toward the edge of a rapid rotating collector. FESEM showed that 950 nm nanofibers aligned on Laminin-coated coverslips (Figure 3A) induced cell bodies to elongate along the axis of the fiber and to extend trailing processes guided by fiber directionality (Figures 3B,C). Directionality quantification can be assessed by processing the image (Supplementary Figure 1D) mathematically by a Fast Fourier Transform (FFT) algorithm (Sachot et al., 2016) represented in a distribution curve (Supplementary Figure 1E) and the full width at half maximum (FWHM). The lower the FMHM, the more oriented the fibers. Interestingly, at the cellular level, TEG3 showed, in some cases, the activity of peripheral lamellipodial waves which bear numerous fine filopodia. This resulted in an increased cell-cell contact and the migration of groups of TEG3 cells over the nanofibers (Figure 3B), as well as single migrations where TEG3 cells extended their long cytoskeleton to align completely following the surface of the PLA nanofiber (950 nm; Figure 3C; Supplementary Videos 1–3). In Supplementary Video 1, the process of adhesion of a single TEG3 cell on a 950 nm PLA nanofiber can be seen. In addition, we can observe, through a high magnification zoom, the interaction of the cell membrane on the migratory front with the nanotopography of PLA nanofiber (Figures 3D,E). Figure 3E details the protrusion of the TEG3 cells when adhering to the fiber. Using immunofluorescence techniques, we were able to show how eGFP-TEG3 cells are capable of associating with fluorescent nanofibers (red; Figure 3F).

### Migratory Properties of TEG3 Cell Over PLA Biofunctionalized SDF-1α Nanofibers on CSPG Substrate

**FIGURE 4:**
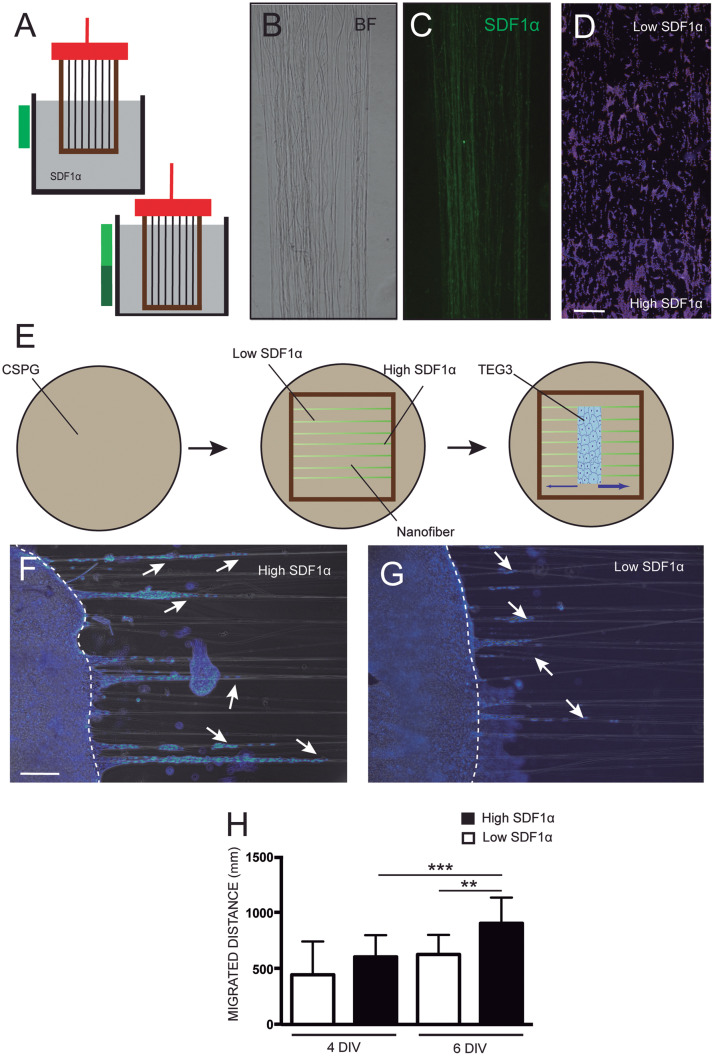
Enhanced adhesion and migration of TEG on SDF-1α functionalized nanofibers. Scheme showing how the paraffin frames with the fibers on the top were dipped into the different aqueous solutions for activation and functionalization **(A)**; optical microscope images showing the maintained structure of the coated fibers in bright-field **(B)**, the homogeneous functionalization of the nanofibers with the chemotactic agent SDF-1α/CXCL12 by linking fluorescent SDF-1α antibodies **(C)**, and **(D)** the concentration of TEG3 cells in two areas with different concentrations of the chemotactic SDF-1α/CXCL12; **(E)** detailed instrumental scheme of the fibers within the paraffin frame laid onto a glass cover-slide coated with inhibitory CSPG where the cells are seeded using a PDMS template container and removed after confluence to let the cells migrate; **(F,G)** immunofluorescence images of the culture on both sides of the initial cell deposit at a time-point of 6 days showing the differences in number and distance migrated (white arrows); and **(H)** quantification of the covered distance by the cells at 4 and 6 days. Kruskal–Wallis test. ***p-value 0,0006; **p-value 0,0026. Scale bars **(C,D)** – 150 μm and **(F,G)** 300 μm.

TEG3 cells growing over CSPG presented a loss of a leading and trailing process and, as well, the presence of membrane protrusions (Supplementary Figures 2C,D) compared to laminin permissive control cells (Supplementary Figures 2A,B). We decided then to create a doubled area with a different concentration of SDF-1α/CXCL12 linked to the surface of the PLA fibers through an EDC/NHS covalent bonding process. First, carboxylate groups had to be generated in a very controlled manner to avoid water meniscus destroying the fibers on the hollow part of the frame. NaOH was selected as the base to break surface PLA chains and induce the creation of carboxylate functional groups (Mateos-Timoneda et al., 2014). The level of concentration of SDF-1α/CXCL12 on the surface was defined by the time of immersion in the solution (Figure 4A). After subsequent controlled immersions in the EDC/NHS and the SDF-1α/CXCL12 solutions, fibers maintained their aligned structure (Figure 4B) and the chemokine was observed with immunofluorescence through the nanofiber (Figure 4C), although with limitations due to the small diameter of the fibers.

Previous to the final test, fibers were electrospun onto conventional glass slides and functionalized with SDF-1α/CXCL12. After sterilization, 2 × 10^5^ TEG3 cells were seeded and cultured for 48 h. Figure 4D shows the preference of TEG3 cells to migrate to high concentrated SDF-1α/CXCL12 functionalized areas rather than areas of lower concentration. In the second set of experiments, the same treatment (with low and high functionalized SDF-1α/CXCL12 areas) was performed for fibers within paraffin frames. However, no functionalization was carried out in the middle of the mats in order to leave a free area for cell deposition. A PDMS mask was placed over the precise location of the non-functionalized area of the fibers (see section “Materials and Methods” for details). 2 × 10^5^ TEG3 cells were seeded in the PDMS hole. After cell adhesion, PDMS was removed and cells were allowed to migrate for between 4 and 6 days (Figure 4E). Cells were observed to advance from the non-functionalized area to the functionalized, especially the highly concentrated areas, as may be observed in Figures 4F–H shows the quantification of the distance migrated by the cells for 4 and 6 days in the effort to find a relevant statistical difference at day 6. The nanofibers then act as a guiding platform for cell migration. On day 6, TEG3 cells migrated an average distance of 625.6 mm ± 175.5 mm (N of 28) for low concentrate SDF-1α/CXCL12 while for high concentrate the average distance migrated was relevantly higher: 903.4 mm ± 230.1 mm (N of 28).

## Discussion

Various studies have indicated that the regenerative properties of OECs seem to be largely associated with their migratory capacities (Wang and Huang, 2012; Roloff et al., 2013), which has led to great interest in improving cell dynamics and survival of OECs after transplantation. Deng et al. (2006) showed that both rat and human OECs migrated relatively short distances, both rostrally and caudally, in animals with spinal cord lesions with a concomitant contralateral hemisection. Smale et al. (1996) reported non-significant cell migration when fetal rat olfactory bulb OECs were transplanted into the brain of damaged adult rats. Also, it has been shown that transplanted OECs migrate shorter distances in injured spinal cord compared to controls (uninjured spinal cord; Gudiño-Cabrera and Nieto-Sampedro, 2000; Collazos-Castro et al., 2005). These results have been confirmed in xenograft experiments using GFP expressing mouse OECs (Ramer et al., 2004).

Determining the molecular and cellular mechanisms by which OECs regulate migratory properties will lead to a better understanding of the role of OECs in regeneration within the olfactory system and will identify how the use of OECs can be optimized for regenerative therapies.

In this study, a parallel polylactic acid PLA electrospun nanofiber with low crystallinity – which improves their flexibility and biodegradability – is proposed. The effect of the diameter was studied and optimized to create a chemotactic concentration increment with a signaling biomolecule such as SDF-1α/CXCL12, which was covalently attached to the fiber surface. Both the topography and the increase in concentration effects were observed to improve the migration and proliferation of the OEC-derived TEG3 cell line over surface coatings with permissive Laminin and, more relevantly, with the inhibitory substrate CSPG. A biodegradable and metabolizable artificial material such as PLA can be processed and modified to introduce specific signaling. This method makes this synthetic polymer comparable to natural materials with the advantage of greater control over processing, and therefore, over the fate of the cell.

Fibers had a high amorphous grade which ensures that the PLA 80/20 is as flexible as possible, although still far from the lack of stiffness of nerve tissue (Supplementary Figure 1A; Sachot et al., 2014). This seems not to be a drawback; as in our previous studies, similar glial cells were able to adhere, proliferate, and even dedifferentiate (Álvarez et al., 2014). FESEM images showed a great affinity of TEG3 cells for pristine PLA fibers without the need for functionalization, evidencing good attachment, adhesion, and alignment on the PLA surface.

We tried to suggest a new strategy for the implantation of those cells in SCI in conjunction with a topographical biomaterial with a signaling chemotactic concentration difference. Biomaterial supports have been proposed in the literature, although to date no one has tried to improve glial migration through a chemotactic integrated signal that bypasses the inhibitory region of the scar. Considering that TEG3 cells express CXCR4, SDF-1α/CXCL12 was an excellent option to enhance not only migration, but also cell adhesion.

Electrospun PLA fibers can be a potent tool to guide TEG3 cells and avoid the inhibition mechanism of the spinal cord environment, as may be observed in the present study. We showed in a previous study that glia can be sensitive to lactate coming from PLA 70/30 fibers and metabolized as an energy source in a glucose-poor environment (Álvarez et al., 2014). However, for this purpose, we needed a less degradable PLA source and a stiffer one that could support aggressive treatment like the process of functionalization shown here. PLA 80/20 meets the requirements to work as a stable bridging platform for neuron migration. As well, the aligned distribution together with the proper thickness work as a perfect tandem for cell alignment and migration. The only thing we needed was to show cells which direction to follow. The possibility of creating a gradient or concentration difference with a chemotactic agent such as SDF-1α/CXCL12 covalently attached by a conventional EDC/NHS chemistry (Sachot et al., 2015) makes the platform ideal for use as a model to bypass the inhibiting CSPG and push the cells toward the concentrated side of the fibers.

The first stages of the research implied the deposition of the fibers and their functionalization onto coverslides. However, that is not practical for a real damaged spinal cord. The idea of using paraffin frames is an attractive option for easy 3D integration of ECM-like fibers. The process here applied has been reproducible and efficient in producing fibers with no background so they can be easily coupled to any 3D tissue, model, device, or material for bridging. They have the option of being stacked, creating different layers of a controlled number of fibers and porosity.

Although the results obtained by several researchers are positive, more research is needed to achieve functional recovery from an SCI. The key phase for axon regeneration is the evolution of the damaged proximal wound into a new growth tip that sums up a regeneration process, which must then cross the lesion through the glial scar and the inflammation to reconnect with its original target (Silver and Miller, 2004). Factors that control the formation of the growth tip and the recovery of axonal growth include the inherent regenerative capacity of neuronal cells, as well as the previously developed multifaceted inhibitory biochemical microenvironment that prevents the growth of the damaged axon. Indeed, they form physical and molecular walls for anatomical and functional recovery. CSPG is a good example of an inhibitory molecule.

Understanding the fundamental mechanism of the type of migration of TEG3 cells from the configuration of a cylindrical topography of diameter around the micrometer, and being able to analyze the spatio-temporal behavior of the cells without subjecting them to a disruption in their structural integrity, were our main concerns. The platform shown here allows us to analyze and characterize the migratory properties of TEG3 cells by microscopy in real-time to obtain a certain migratory pattern. Preliminary, but not conclusive, results indicate that TEG3 cells are capable of migrating collectively, but with free disposition. A lack of stable cell-cell junctions was observed (see Supplementary Video 2 in the Supplementary Data) during the migratory process; however, transitory physical interactions were observed, and perhaps they could also be molecular, which would allow free movement within a coordinated migration.

The approach presented here needs to be tested in a real inhibitory environment. However, the process can be further optimized, for example with the combination of SDF-1α/CXCL12 surface concentration increments and TEG3 cell lines expressing the ectodomain of the Nogo receptor 1 (NgR1), an antagonist of MAIs. Finally, the application of neurons and determination of whether the growth tip and the recovery of axonal growth can be controlled within the inherent regenerative capacity of neuronal cells need to be examined. We are convinced that TEG3 cells in our platform can form physical and molecular walls for anatomical and functional recovery.

The next steps involve the addition of other interesting experiments such as inhibiting the CXCR4 co-receptor in the TEG3 cell line as a proof of concept to evidence that molecular interactions through this receptor-ligand complex are fundamental for the growth of OECs over CSPGs; as well, co-cultures with neurons are envisaged, to show that OECs higher levels of directionality and migration can positively influence the neurons growth and their axonal extension; and finally, explore the adhesion molecules/proteins, or other overexpressed markers that might explain the increased cell adhesion and migration. However, identification of the main targets and the increase of the dimension and the number of cells adhered to the fibers is needed and a challenge.

## Conclusion

Poly(l/dl-lactic acid electrospun fibers with a covalently linked signaling SDF-1α/CXCL12 surface increment act as an efficient bridge for TEG3 cell migration. Although fiber stiffness is far from the natural stiffness of the nerve tissue, topographical morphology, and the possibility to effectively link an amino-containing biomolecule with time-dependent dip-coating using a well-known chemistry method, opens the way to developing more suitable platforms involving biomaterials. TEG3 cells migrate onto the fiber despite being surrounded by an inhibitory environment of CSPG. Real-time migration analysis and characterization over a nanostructure pattern are easily achieved without the need for micronanufacture with sophisticated instrumentation techniques. Indeed, we increase the migration of the cells over inhibitory substrates (as happens after SCI) on compatible fibers most probably their immunomodulatory effects can be preserved for longer times to enhance axon regrowth of lesioned neurons. However, these are the next step not only *in vitro* but also *in vivo.* Further experiments are needed to explore the mechanisms behind OECs migration and adhesion, which would be of paramount importance to enhance surfaces in terms of healing efficiency.

## Data Availability Statement

The raw data supporting the conclusions of this article will be made available by the authors, without undue reservation.

## Author Contributions

OC, EE, and JR conceived the project. OC, AL-M, DR, and AM-A implemented and carried out the experiments, characterized the outcomes, and analyzed the data. OC, AL-M, DR, and JR wrote the manuscript. OC, EE, and JR provided the funding and the needed infrastructure. All authors contributed to the article and approved the submitted version.

## Conflict of Interest

The authors declare that the research was conducted in the absence of any commercial or financial relationships that could be construed as a potential conflict of interest.

## Supplementary Material

Resulted plots form mechanical and thermal characterization; Electronic and optical fluorescence microscope images of TEG3 cells both on adhesive and inhibitory coatings.

Supplementary Video 1 TEG3 cells running over 950 nm fibers.

Supplementary Video 2 TEG3 cells migration front over 950 nm fiber.

Supplementary Video 3 TEG3 cells advance over a 950 nm fiber in a CSPG inhibtory coating.
