# Supplementary material for: Chemotactic TEG3 Cells’ Guiding Platforms Based on PLA Fibers Functionalized With the SDF-1α/CXCL12 Chemokine for Neural Regeneration Therapy
Source: Front Bioeng Biotechnol. 2021 Mar 22;9:627805. doi: 10.3389/fbioe.2021.627805 (PMC8019790; doi:10.3389/fbioe.2021.627805)
Supplement: Supplementary file 4 [file Data_Sheet_1.pdf]

Supplementary Material

SDF-1α /CXCL12 concentration gradients covalently fixed on PLA fibers guide migration of olfactory ensheathing cell (OEC) over inhibitory substrate chondroitin sulfate proteoglycans (CSPG).

Oscar Castaño [1,2,3,4,*], Ana López-Mengual [6,7,8,9,*], Diego Reginensi [10,11,*], Andreu Matamoros-Angles[6,7,8,9,12], Elisabeth Engel [2,3,5] ✉, José Antonio del Rio[6,7,8,9] ✉.

Supplementary Figures

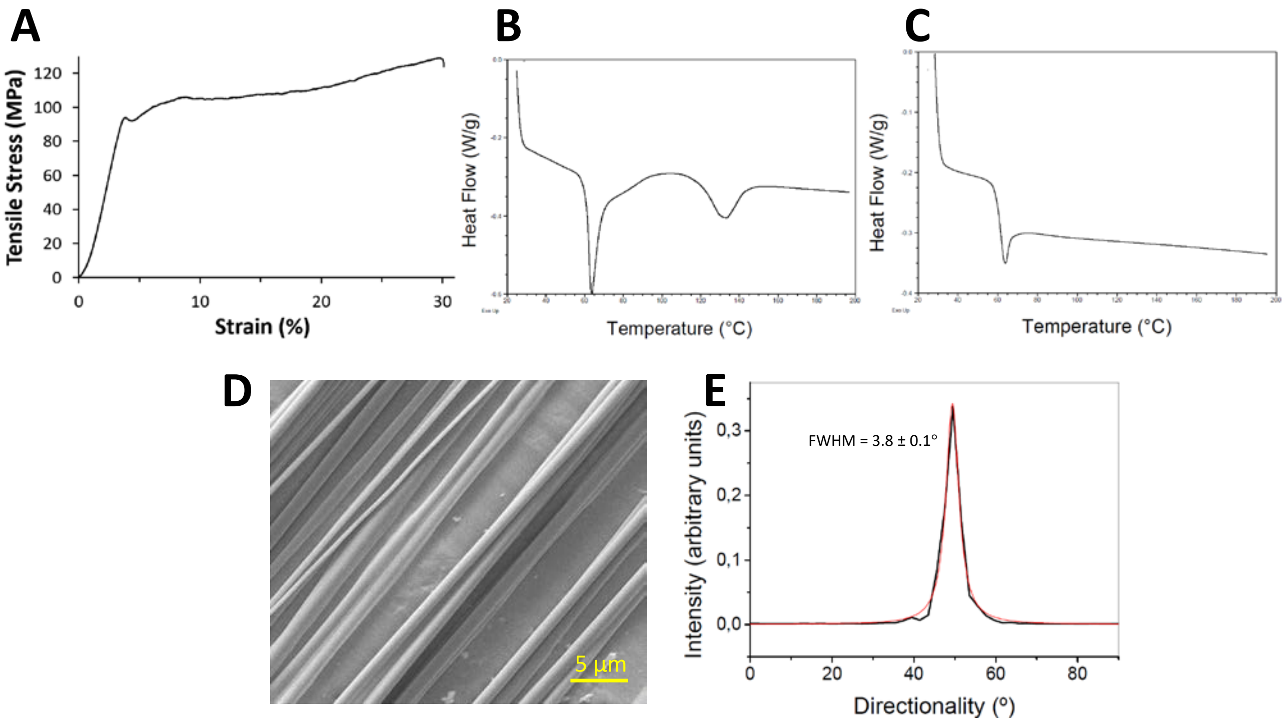

Supplementary Figure 1. A) Typical mechanical analysis of an aligned electrospun mat; B) DSC first cycle analysis of the electrospun fibers for the assessment of the processing history of the material and showing a melting point at T_m = 132.8ºC; C) DSC second heating cycle showing the glass transition temperature at T_g = 59.1ºC. D) Typical FESEM image of 950nm fibers E) FFT analysis of the directionality showing a FWHM of 3.8 ± 0.1°, which shows an uniaxial alignment of the fibers.

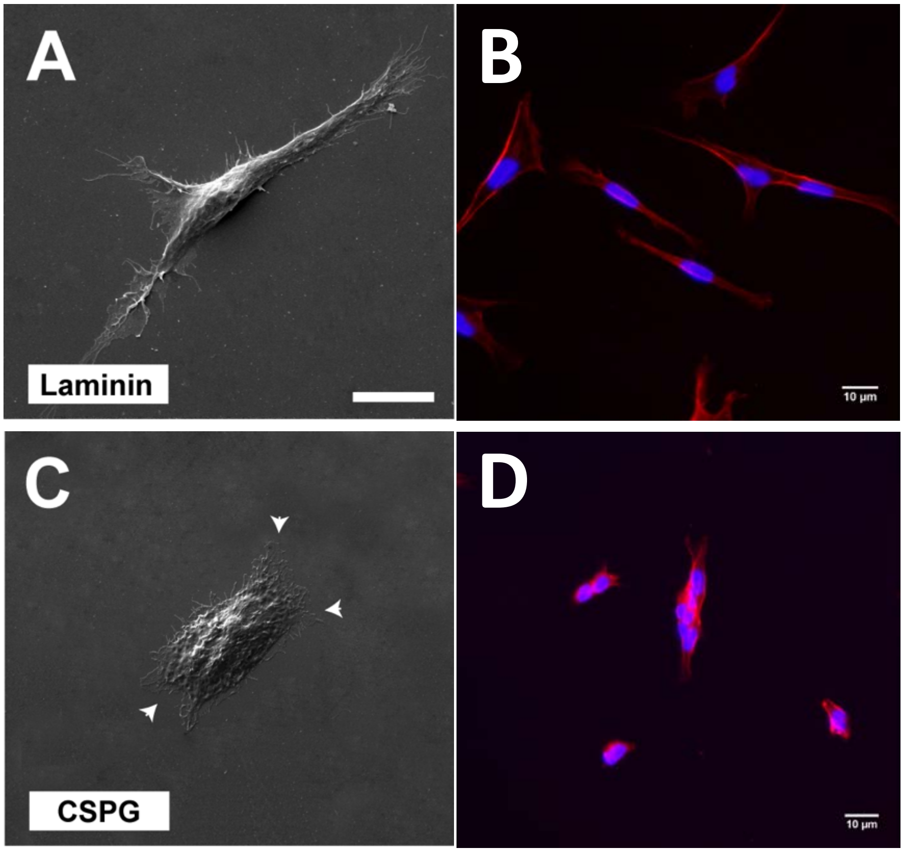

Supplementary Figure 2: FESEM and DAPI/phalloidin stained fluorescence images of fixed TEG3-OEG cells over coatings of (A and B) adhesive laminin coating and (C and D) inhibiting CSPG to observe their adhesion behavior and shape.
